# Acai Supplementation and Resistance Training: A Preliminary Study of the Effects on Liver Parameters in Hypertensive Rats

**DOI:** 10.3390/life16071056

**Published:** 2026-06-24

**Authors:** Ângela Quinelato Oliveira, Pilar Barbosa de Meireles, Willian Cruz Ribeiro, Luciano Bernardes Leite, Pedro Forte, Alexandra Malheiro, Pedro Afonso, Anselmo Gomes de Moura, Denise Coutinho de Miranda, Luiz Otávio Guimarães-Ervilha, Mariana Machado-Neves, Antônio José Natali, Victor Neiva Lavorato

**Affiliations:** 1 Department of Physical Education and Nutrition, Governador Ozanam Coelho University Center, Ubá 36506-022, MG, Brazil; angela.quinelato.o@gmail.com (Â.Q.O.); pimeireless@gmail.com (P.B.d.M.); wzenaide29@gmail.com (W.C.R.); anselmo.moura@unifagoc.edu.br (A.G.d.M.); denise.miranda@unifagoc.edu.br (D.C.d.M.); victor.lavorato@ifms.edu.br (V.N.L.); 2 Laboratory of Exercise Biology, Federal University of Viçosa, Viçosa 36590-000, MG, Brazil; anatali@ufv.br; 3 Department of Sports, Instituto Politécnico de Bragança, 5300-253 Bragança, Portugal; 4 Research Center for Active Living and Wellbeing (LiveWell), Instituto Politécnico de Bragança, 5300-253 Bragança, Portugal; alexandra.malheiro@iscedouro.pt; 5 Department of Sports, Higher Institute of Educational Sciences of the Douro, 4560-708 Penafiel, Portugal; pmvafonso@gmail.com; 6 Biosciences Higher School of Elvas, Polytechnic Institute of Portalegre, 7300-110 Portalegre, Portugal; 7 Departament of Biological Sciences, Federal University of Viçosa, Viçosa 36590-000, MG, Brazil; luiz.ervilha@ufv.br (L.O.G.-E.); mariana.mneves@ufv.br (M.M.-N.); 8 Federal Institute of Mato Grosso do Sul, Ponta Porã Campus, Ponta Porã 79909-000, MS, Brazil

**Keywords:** hypertension, hepatocytes, Euterpe oleracea, resistance training, spontaneously hypertensive rats

## Abstract

Systemic arterial hypertension (SAH) is a multifaceted condition marked by sustained elevations in arterial blood pressure. Its occurrence is closely related to alterations in target organs, such as the liver. Non-pharmacological treatments have been proposed for these effects. Thus, the aim of this study was to investigate the effects of açaí supplementation and resistance training, applied individually or in combination, on blood pressure and liver structural parameters. An experimental, quantitative, and longitudinal study was conducted using young Wistar rats (~60 days old) and spontaneously hypertensive rat (SHR) strains. Fifty rats were divided into five experimental groups: Wistar Control (C), Hypertensive Control (H), Hypertensive Trained (HT), Hypertensive Açaí-Supplemented (HA), and Hypertensive Trained plus Açaí Supplementation (HAT). Each group consisted of ten animals. Subsequently, analyses were performed for the antioxidant capacity and proximate composition of the açaí pulp, systolic blood pressure assessment, and histological evaluation of the liver. The açaí used exhibited high antioxidant capacity. At the end of the experimental period, the trained groups increased their maximal load carried, along with a reduction in systolic blood pressure in all treated groups. Açaí supplementation resulted in lower relative liver mass compared with the H group. The hypertensive condition promoted extracellular matrix expansion and a reduction in hepatocyte proportion. Both interventions attenuated these effects, and the combined treatment (HAT) produced the greatest improvement, indicating an additive response. Hypertension also elevated hepatic glycogen concentration, and the treatments reduced this alteration. It is concluded that açaí supplementation and resistance training could promote positive adaptations in the liver of hypertensive animals.

## 1. Introduction

Systemic arterial hypertension (SAH) is a multifaceted condition whose development is driven by genetic, epigenetic, environmental, and psychosocial contributors [1]. The condition is characterized by sustained elevations in arterial pressure, with systolic blood pressure ≥ 140 mmHg and/or diastolic blood pressure ≥ 90 mmHg [1]. Besides being the main risk factor for cardiovascular diseases, SAH is associated with the development of a wide range of chronic conditions and is responsible for 10.8 million deaths worldwide [2,3]. It is a predominantly asymptomatic and silent disorder, whose progression may lead to structural and functional alterations in various target organs over time [4].

To investigate the pathophysiological mechanisms of hypertension, several experimental models have been employed, with the Spontaneously Hypertensive Rat (SHR) standing out as one of the most established. This model develops hypertension spontaneously and progressively, reproducing clinical and hemodynamic characteristics like those observed in humans [5]. These animals exhibit sympathetic nervous system hyperactivity, endothelial dysfunction, renin–angiotensin–aldosterone system activation, cardiovascular remodeling, and increased oxidative stress and inflammation [5]. In addition, the SHR presents experimental advantages such as low cost, small body size, and a short life cycle, which favor its wide use in preclinical studies [6]. The similarity between the progression of hypertension in this model and in humans allows for a consistent assessment of the systemic repercussions resulting from chronic hypertension.

Among the organs affected by hypertension, the liver has been gaining prominence [7]. Worldwide, chronic liver diseases affect nearly 38% of the population and constitute a major public health concern. Their clinical presentation ranges from mild hepatic abnormalities to severe conditions, including fibrosis, cirrhosis, and hepatocellular carcinoma [8,9].

The progression of hepatic dysfunction is associated with the activation of fibrotic pathways, especially in subjects with metabolic and cardiovascular comorbidities, including hypertension. This process favors extracellular matrix accumulation and is accompanied by a persistent inflammatory state marked by impaired cell signaling, greater apoptosis, and elevated oxidative stress, thereby aggravating liver damage [10,11].

Non-pharmacological strategies have been extensively explored as alternatives to attenuate liver alterations associated with hypertension, particularly nutritional interventions and exercise-based approaches. In this context, açaí (Euterpe oleracea Mart.) has gained attention because it is a rich source of bioactive compounds, notably anthocyanins, which are recognized for their antioxidant and vasoprotective effects [12,13]. Evidence suggests that açaí consumption may contribute to both the prevention and management of hypertension, while also promoting hepatoprotection through the reduction in inflammatory mediators and the enhancement of endogenous antioxidant defenses [14,15,16]. Its administration has been associated with the prevention and treatment of hypertension, in addition to exerting a hepatoprotective effect by reducing pro-inflammatory markers and increasing endogenous antioxidant defense [14,15,16]. In parallel, resistance training, that is, the modality that consists of effort performed against a force generated by a given resistance, with the aim of increasing muscle strength, has also been identified as an effective intervention, as it reduces blood pressure, decreases peripheral vascular resistance, and stimulates the activity of antioxidant enzymes [17,18]. Furthermore, recent evidence demonstrates its hepatoprotective potential, showing, for example, a reduction in hepatic lipid accumulation, a decrease in liver enzymes, and an attenuation of insulin resistance after 12 weeks of intervention [19,20].

However, despite the beneficial effects already attributed to açaí and resistance training individually, the possible combined effects of these interventions on hepatic parameters in SHR animals remain unknown. Thus, it becomes pertinent to investigate the joint action of these non-pharmacological approaches in the context of hepatic impairment associated with hypertension. Therefore, the aim of this study was to investigate the effects of açaí supplementation and resistance training, applied individually or in combination, on systolic blood pressure and hepatic structural parameters including extracellular matrix proportion, hepatocyte percentage, and glycogen accumulation in SHR.

## 2. Materials and Methods

### 2.1. Animals

Young rats (≈60 days) of the Wistar and SHR strains were used, housed in polyethylene cages with up to five animals per unit, and maintained with ad libitum access to commercial chow and water. Environmental conditions were controlled, with a temperature of 22 ± 2 °C and a 12/12 h light/dark cycle. The experimental period lasted 22 weeks, counted from the arrival of the animals at the UNIFAGOC Animal Facility, and all animals were weighed weekly throughout the protocol. A total of 50 rats were assigned to five experimental groups: Wistar Control (C), Hypertensive Control (H), Hypertensive Trained (HT), Hypertensive Açaí-Supplemented (HA), and Hypertensive Trained plus Açaí Supplementation (HAT), with ten animals in each group. All experimental procedures were conducted in accordance with the approval granted by the Ethics Committee for Animal Use of the Governador Ozanam Coelho University Center (protocol no. 02/2022). Euthanasia was performed by decapitation 48 h after the final resistance training session and/or açaí administration.

### 2.2. Acquisition and Preparation of Açaí Pulp

The acquisition and preparation of the açaí pulp followed standardized procedures. Pulp previously subjected to the pasteurization process was used, selecting a preservative-free brand (Layne Agroindústria, Ubá, Brazil—energy value per 70 g serving = 175 kcal, carbohydrates = 26 g, protein = 2.8 g, fat = 8.4 g, fiber = 12 mg, vitamin C = 23 mg), which remained stored in a freezer until use. Prior to use, the pulp was allowed to thaw at room temperature and then passed through filter paper. The resulting filtrate was subsequently used both for animal administration and for the determination of proximate composition, phytochemical profile, and antioxidant capacity.

### 2.3. Açaí Management

Açaí administration was performed daily at 7:30 a.m. by gavage for the animals belonging to the supplemented groups. Based on the protocol established by Guerra et al. [21], açaí supplementation was provided at a dose of 3 g per kilogram of body weight. The pulp, previously filtered through filter paper (Whatman no. 1, Maidstone, UK), was administered without dilution, following the protocol employed in previous studies [16]. Considering that the maximum administrable volume in rodents is 1 mL of solution for every 100 g of body weight, which may reach up to 2 mL when dealing with aqueous solutions, the final volume to be administered was calculated using the formula: (body weight × 3)/1000.

### 2.4. Antioxidant Capacity

The antioxidant capacity of the filtered pulp was determined using the 2,2-diphenyl-1-picrylhydrazyl (DPPH) radical method, which is based on the transfer of electrons from antioxidant compounds to the stable DPPH radical [22]. The antioxidant activity of the açaí pulp was evaluated using the DPPH radical scavenging assay. Briefly, 0.1 mL of pulp was mixed with 3.9 mL of a 60 μmol/L DPPH solution prepared in 80% methanol. After thorough mixing, the reaction mixtures were incubated for 30 min in the dark at room temperature. Control samples were prepared under identical conditions, substituting the pulp with distilled water. Absorbance was subsequently measured at 515 nm using a spectrophotometer (Biospectro^®^, Curitiba, PR, Brazil), with 80% methanol serving as the blank. Quantification was based on a calibration curve generated with Trolox (6-hydroxy-2,5,7,8-tetramethylchroman-2-carboxylic acid; Sigma-Aldrich^®^, St. Louis, MO, USA) at concentrations ranging from 100 to 800 μmol/L. Antioxidant capacity was expressed as Trolox Equivalent Antioxidant Capacity (TEAC; μmol Trolox equivalents per gram of açaí). The pulp contained 8.02 ± 0.11 mg GAE/g of total phenolic compounds and exhibited an antioxidant capacity of 18.19 ± 0.90 μmol Trolox/g.

### 2.5. Centesimal Composition

Analyses of the proximate composition were performed at the UFV Laboratory of Food Analysis and Nutritional Biochemistry in accordance with Adolfo Lutz Institute guidelines. Sample moisture was measured through oven drying at 105 °C to constant weight. Lipid content was determined using the Bligh–Dyer extraction procedure with chloroform and methanol as solvents. For ash determination, samples were subjected to combustion in a muffle furnace at temperatures ranging from 550 to 660 °C. Proteins were quantified using the Kjeldahl method, with protein content calculated from the total nitrogen amount, employing a conversion factor of 6.25. The carbohydrate fraction was obtained by difference, considering the values already determined for moisture, lipids, proteins, and ash.

The proximate composition of the açaí pulp revealed a high moisture content (93.53 ± 1.46%). On a dry-weight basis, 12.68 ± 1.27% protein, 50.70 ± 0.28% lipids, 21.47 ± 1.12% fiber, 3.78 ± 0.02% ash, and 11.37% carbohydrates were identified.

### 2.6. Systolic Blood Pressure

Systolic blood pressure (SBP) was assessed non-invasively by adapted tail-cuff plethysmography. For the measurements, animals were placed in restraining chambers and passively warmed at 29–32 °C for 10 min to promote dilation of the caudal artery. Subsequently, a pressure cuff was positioned approximately 3 cm from the tail tip. After pulse detection, the cuff was inflated and SBP values were recorded.

### 2.7. Resistance Training Protocol

The resistance training protocol originally described by Hornberger and Farrar [23] was adapted to meet the specific requirements of this study. The rats first underwent a two-week familiarization period, with three sessions per week, during which they were gradually introduced to resistance exercise. In this stage, the animals practiced climbing a vertical ladder (1.1 × 0.18 m; 2 cm spacing between rungs; 80° incline) while carrying an external load attached to the tail. After reaching the top, they rested for 120 s. This procedure was repeated until each rat was able to climb the ladder voluntarily three consecutive times without stimulation. During the second week, the animals performed two to three voluntary climbs per session using a 20 g weight secured to the proximal tail with adhesive tape.

Upon completion of the adaptation phase, animals underwent a maximal load test as previously described [24]. The test was repeated every month to adjust the training loads and once more at the end of the experimental period to determine strength adaptations. The maximum load achieved served as a measure of tolerance to physical effort. Resistance training began immediately after the load test and was structured into three mesocycles. Training volume and intensity progressed throughout the protocol, starting with five sets at 60% of the maximal load and advancing to twelve sets at 75% of the maximal load carried [25].

### 2.8. Histological Analysis of the Liver

Fragments of hepatic tissue were fixed in 10% Carlsson’s formalin for 48 h. The fragments were then dehydrated in increasing ethanol series (80%, 90%, 95%, and absolute), remaining for 30 min in each concentration, followed by immersion in resin for 24 h. After this stage, the samples were embedded in resin with hardener and kept in an oven at 60 °C for 48 h for complete polymerization. Tissue blocks were cut into 5 µm sections with a rotary microtome (Spencer, model 19459, Buffalo, NY, USA) and stained with H&E. Images were acquired under a light microscope (Olympus BX-50, Tokyo, Japan) equipped with a digital imaging system (Olympus Q Color-3, Tokyo, Japan), with 50 random fields collected per animal. Morphometric analysis was performed using a grid of 266 intersection points superimposed on each image, allowing estimation of the relative proportions of hepatocytes and extracellular matrix through point counting. The values obtained from the 50 images were averaged per animal prior to statistical analysis, resulting in one representative value per animal. Histological and morphometric analyses were performed by a blinded evaluator, who was unaware of the experimental group allocation during image acquisition. All analyses were performed using the Image-Pro Plus 4.5 software (Media Cybernetics, Silver Spring, MD, USA). For histological analyses, a randomly selected subset of animals per group was processed, resulting in 6 to 8 animals per group.

Another portion of the samples, also sectioned at 5 µm, was stained with periodic acid–Schiff (PAS) for the assessment of hepatic glycogen accumulation. The slides were visualized and recorded following the same procedure used for HE staining. For quantification, fifty random images per animal were selected, and the resulting values were averaged per animal prior to statistical analysis. Areas of glycogen accumulation were delineated in the Image-Pro Plus 4.5 software, which subsequently performed the counting and automatic calculation of the corresponding percentage.

### 2.9. Statistical Analysis

The assumption of normality was examined with the Shapiro–Wilk test. Parametric data were compared by one-way ANOVA and Tukey’s post hoc test, whereas nonparametric variables were analyzed using the Kruskal–Wallis test with Dunn’s multiple-comparison correction. Additionally, a two-way ANOVA with resistance training and açaí supplementation as between-subject factors was performed for hepatic outcome variables to assess the interaction between interventions. For histological analyses, data were averaged per animal prior to statistical testing. To compare body mass, physical capacity, and systolic blood pressure between the initial and final moments, the paired Student’s *t*-test or the Wilcoxon test was employed, according to data distribution. Quantitative data are expressed as mean ± standard deviation, whereas categorical results are presented as percentages. Statistical analyses were carried out in GraphPad Prism 10.0, with significance set at *p* < 0.05.

## 3. Results

**Figure 1:**
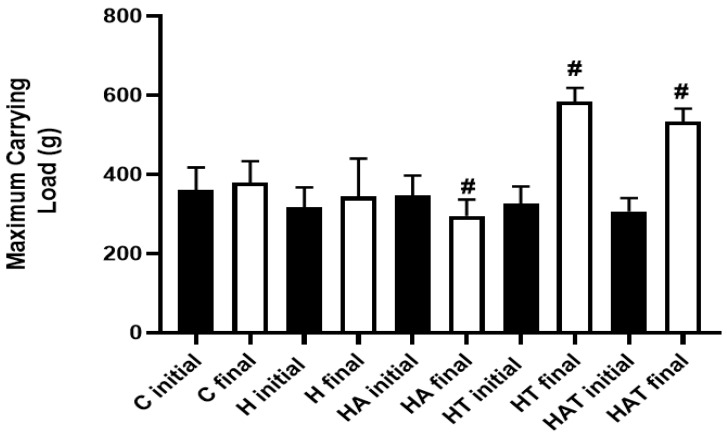
Initial and final load carried by the experimental groups. Values are presented as mean ± standard deviation based on 8–10 animals per group. C, Control. H, Hypertensive. HA, Hypertensive + Açaí. HT, Hypertensive + Resistance Training. HAT, Hypertensive + Açaí + Resistance Training. #, difference from the initial moment within the same group.

Figure 1 presents the data related to the physical test over the intervention weeks. It was observed that the Hypertensive Açaí group showed a significant reduction in the load carried at the end of the experimental period (HA initial = 347.4 ± 49.79 vs. HA final = 295.3 ± 41.22; *p* = 0.0037). In contrast, the groups subjected to resistance training demonstrated a marked increase in the load carried at the end of the intervention (HT initial = 325.5 ± 44.29 vs. HT final = 584.4 ± 34.03; HAT initial = 306.8 ± 33.58 vs. HAT final = 533.8 ± 32.32; in all comparisons, *p* < 0.0001).

Table 1 presents the results for initial and final body mass, as well as initial and final systolic blood pressure. It was observed that, at the beginning of the experiment, the Control group showed greater body mass compared with the other groups. Additionally, all hypertensive groups exhibited a significant increase in body mass at the end of the intervention period compared with initial values (*p* < 0.0001). Regarding systolic blood pressure, a significant reduction was observed at the end of the intervention in the Hypertensive Trained, Hypertensive Açaí, and Hypertensive Açaí Trained groups (*p* = 0.0292; *p* = 0.0016; *p* < 0.0001, respectively).

Table 2 presents the data related to absolute and relative liver mass. Regarding absolute mass, the Hypertensive Açaí Trained group showed a lower value compared with the Control group (*p* = 0.043). With respect to relative liver mass, a significant increase was observed in the Hypertensive group compared with the Control group (*p* = 0.0006). Additionally, the Hypertensive Açaí and Hypertensive Açaí Trained groups showed a reduction in relative liver mass when compared with the Hypertensive group (*p* = 0.0009; *p* = 0.0054, respectively).

**Figure 2:**
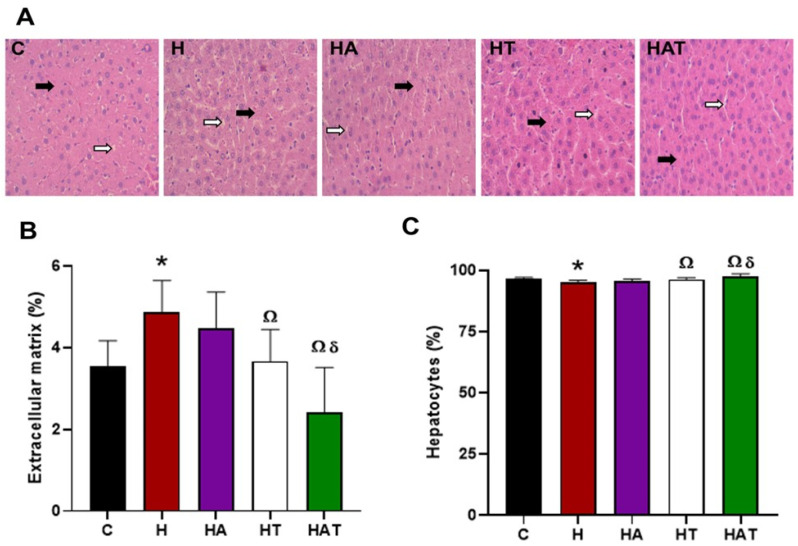
Histological parameters in the liver. (**A**) Representative microscopic photographs of liver sections from experimental animals. (**B**) Proportion of extracellular matrix. (**C**) Proportion of hepatocytes. Black arrows indicate hepatocytes. White arrows indicate extracellular matrix. Values are presented as mean ± standard deviation based on 8–10 animals per group. C, Control. H, Hypertensive. HA, Hypertensive + Açaí. HT, Hypertensive + Resistance Training. HAT, Hypertensive + Açaí + Resistance Training. *, difference from group C. Ω, difference from group H. δ, difference from groups HA and HT.

Figure 2 presents the histological data of livers stained with HE. Statistically significant differences were observed both in the percentage of extracellular matrix (*p* < 0.0001) and in the percentage of hepatocytes (*p* < 0.0001). Hypertension promoted an increase in extracellular matrix (C = 3.53 ± 0.64% vs. H = 4.86 ± 0.78%) and a reduction in the proportion of hepatocytes (C = 96.47 ± 0.64% vs. H = 95.14 ± 0.78%). Administration of the interventions, either individually or combined, resulted in a reduction in the extracellular matrix (H = 4.86 ± 0.78% vs. HA = 4.46 ± 0.90%; HT = 3.67 ± 0.78%; HAT = 2.41 ± 1.11%) and an increase in the percentage of hepatocytes (H = 95.14 ± 0.78% vs. HA = 95.46 ± 0.88%; HT = 96.29 ± 0.74%; HAT = 97.51 ± 1.06%). Two-way ANOVA revealed no significant interaction between resistance training and açaí supplementation for extracellular matrix (F = 1.98, *p* = 0.170) or hepatocyte proportion (F = 2.44, *p* = 0.128), indicating that the interventions acted independently. Notably, the HAT group showed the greatest magnitude of improvement, consistent with additive effects of the combined interventions.

**Figure 3:**
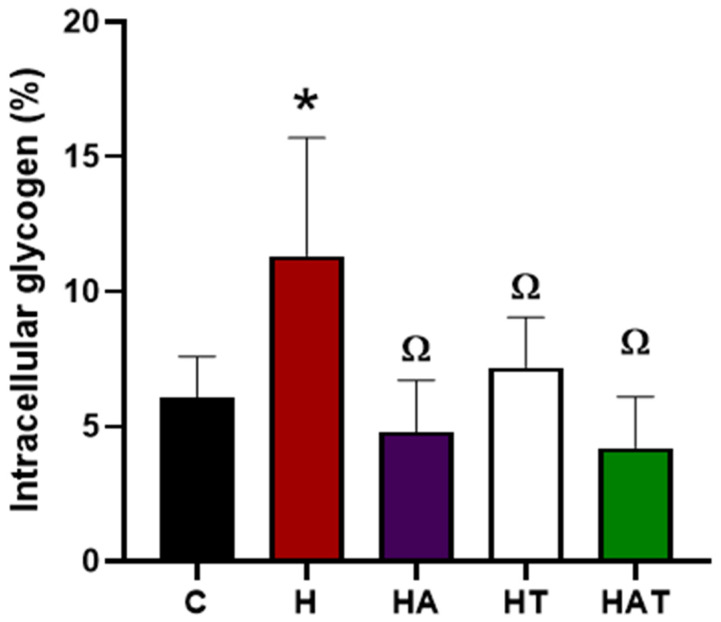
Proportion of intracellular glycogen in the liver of experimental animals. Values are presented as mean ± standard deviation based on 8–10 animals per group. C, Control. H, Hypertensive. HA, Hypertensive + Açaí. HT, Hypertensive + Resistance Training. HAT, Hypertensive + Açaí + Resistance Training. *, difference from group C. Ω, difference from group H.

Figure 3 presents the histological data of livers stained with PAS. The analyses revealed statistically significant differences among the groups (*p* < 0.0001). It was observed that hypertension increased the percentage of hepatic glycogen compared with the Control group (C = 6.08 ± 1.52% vs. H = 11.31 ± 4.38%). Furthermore, the interventions were able to attenuate this effect, reducing hepatic glycogen accumulation (H = 11.31 ± 4.38% vs. HA = 4.79 ± 1.92%; HT = 7.18 ± 1.86%; HAT = 4.64 ± 2.23%). Two-way ANOVA revealed a significant interaction between resistance training and açaí supplementation on hepatic glycogen (F = 4.46, *p* = 0.043), suggesting that the combined intervention produced a greater reduction in glycogen accumulation beyond the effects of each intervention individually.

## 4. Discussion

The present study investigated the effects of açaí supplementation and resistance training on hepatic parameters in hypertensive animals. Overall, it was observed that training increased load-carrying capacity, while all groups showed an increase in body mass throughout the intervention. Hypertension elevated systolic blood pressure; however, both training and açaí supplementation were able to attenuate this increase. In the liver, hypertension promoted greater relative mass, increased extracellular matrix, reduced proportion of hepatocytes, and increased glycogen accumulation, effects that were attenuated by individual treatments and by the combined intervention.

Açaí has a high concentration of polyphenolic compounds, such as anthocyanins, which confer strong antioxidant capacity [26,27]. The composition of the pulp used in this study showed a substantial phenolic content and considerable antioxidant capacity, comparable to or exceeding that reported in other studies [21,28,29], reinforcing the quality of the material provided to the animals. These characteristics may explain the effects observed on systolic blood pressure, since açaí extracts have been shown in previous studies to demonstrate vasodilatory activity possibly mediated, in part, by increased nitric oxide bioavailability and reduced reactive oxygen species, although these mechanisms were not directly measured in the present study [30,31,32].

Resistance training, in turn, is widely recognized as an effective non-pharmacological intervention for reducing blood pressure in hypertensive individuals and animal models, possibly acting through mechanisms such as increased nitric oxide production, reduced sympathetic activation, and decreased peripheral vascular resistance, as previously described in the literature [33,34,35]. In the present study, the trained groups showed a significant increase in load-carrying capacity, accompanied by a reduction in systolic blood pressure findings consistent with the literature [25,36]. Although açaí has antioxidant and anti-inflammatory properties described in the literature, its effects may be more related to metabolic and cardiovascular protection than to a direct increase in muscle strength [21]. Thus, in the absence of resistance training stimulus, sufficient muscle adaptations did not occur to maintain or increase maximum load. The significant decline observed in the HA group may reflect natural physical deconditioning in hypertensive animals not subjected to exercise training, rather than a direct adverse effect of açaí supplementation, though this warrants further investigation.

In the liver, hypertension resulted in greater extracellular matrix and a lower proportion of hepatocytes. Although the absolute differences in extracellular matrix percentage were modest, early and subtle changes in hepatic architecture have been shown to carry biological relevance in hypertensive models, as they may precede more pronounced structural deterioration over time [37,38]. These findings are consistent with the literature, which identifies hypertension as a risk factor for liver changes associated with chronic inflammation, insulin resistance, and activation of the renin-angiotensin system, particularly via angiotensin II. Although these molecular pathways were not directly assessed in the present study, the structural alterations observed in the H group are consistent with this body of evidence [39,40,41]. Previous studies have shown that SHR animals exhibit greater hepatic oxidative stress, reduced antioxidant enzyme activity, and alterations in hepatic extracellular matrix remodeling [37,38], which reinforces the findings of the present work.

Açaí supplementation and resistance training attenuated the hepatic alterations observed, reducing extracellular matrix and increasing the percentage of hepatocytes. These effects are consistent with studies showing that açaí and resistance training reduce steatosis, inflammation, and liver mass in models of metabolic injury [15,16,21,28]. The most pronounced impact of resistance training on the evaluated parameters possibly occurred due to metabolic and vascular adaptations induced by regular physical exercise, promoting cellular preservation [28]. The combination of exercise and açaí, as observed by De-Bem et al. [42], may combine their individual benefits, which was consistent with the present study, as the HAT group showed the greatest histological improvements, reflecting additive effects of both interventions.

Regarding hepatic glycogen, an increase was observed in hypertensive animals, followed by a reduction in the treated groups. Hypertension is associated with greater glycogen synthase activity and alterations in glucose metabolism [43]. Resistance training, as demonstrated by Santos et al. [44], can reduce hepatic glycogen stores by promoting greater energy utilization and improving metabolic function. However, the literature presents divergent results regarding hepatic glycogen behavior in response to exercise or açai [42,45], and methodological differences may explain part of these inconsistencies. Although the increase in glycogen observed in the hypertensive group may seem paradoxical, alterations in insulin sensitivity, glycogen synthase activity, and hepatic remodeling may contribute to this finding [46]. Notably, a significant interaction between resistance training and açaí supplementation was observed for hepatic glycogen, suggesting that the combined intervention produced a greater reduction in glycogen accumulation beyond the effects of each intervention individually. As metabolic parameters such as insulin, glucose tolerance, and HOMA-IR were not assessed, the precise mechanism underlying this interaction remains unclear, representing a limitation of this work.

Finally, some limitations should be considered. The absence of molecular markers such as hepatic oxidative stress indicators, inflammatory cytokines, and renin-angiotensin system components limits mechanistic interpretations, while the lack of serum hepatic function markers (ALT, AST, ALP, GGT, albumin, and bilirubin) and metabolic parameters (insulin, glucose tolerance, and HOMA-IR) restricts the functional interpretation of the structural findings. Additionally, food and caloric intake were not recorded, so potential differences in energy consumption between groups cannot be excluded as contributing factors to the observed results in body mass, liver mass, and glycogen. The biological relevance of the modest extracellular matrix changes should also be interpreted cautiously, as HE-based morphometry may not capture the full extent of early hepatic remodeling. Finally, studies simultaneously evaluating resistance training and açaí supplementation in the context of hypertension and liver health remain scarce, indicating the need for further investigations.

## 5. Conclusions

It is concluded that açaí, together with resistance training, can promote positive adaptations, such as reduced systolic blood pressure at the end of the intervention period, decreased relative liver mass in the treated groups, reduced extracellular matrix percentage, and increased hepatocyte proportion. Furthermore, the combination of both treatments resulted in the greatest overall improvements, with additive effects observed for extracellular matrix and hepatocyte proportion, and a significant interaction effect identified for hepatic glycogen, suggesting a combined benefit beyond individual interventions for this parameter.

Although the present study did not evaluate fibrotic parameters through specific staining methods such as Sirius Red or Masson trichrome, the proportional changes in extracellular matrix observed by HE staining may reflect early structural modifications of biological relevance.

## Figures and Tables

**Table 1:** Initial and final body mass and initial and final systolic blood pressure of the experimental groups.

	C	H	HA	HT	HAT
Initial Body Mass (g)	402.3± 44.28 ^σ^	288.6 ± 11.97	303.7 ± 20.70	298.3 ± 38.72	289.4 ± 21.94
Final Body Mass (g)	428.1 ± 43.00 ^σ^	346.6 ± 26.41 ^#^	360.5 ± 25.96 ^#^	351.7 ± 39.65 ^#^	346.0 ± 17.20 ^#^
Initial SBP (mmHg)	116.9 ± 13.08 ^σ^	198.9 ± 18.33	196.8 ± 12.30	199.4 ± 11.58	201.0 ± 11.01
Final SBP (mmHg)	116.1 ± 3.53 ^σ^	208.3 ± 12.29	179.2 ± 13.32 ^#^	172.3 ± 14.65 ^#^	166.3 ± 14.96 ^#^

Values are presented as mean ± standard deviation based on 8–10 animals per group. C, Control. H, Hypertensive. HA, Hypertensive + Açaí. HT, Hypertensive + Resistance Training. HAT, Hypertensive + Açaí + Resistance Training. ^#^, difference from the initial moment within the same group. ^σ^, difference from the other groups.

**Table 2:** Liver mass and relative liver mass.

	C	H	HA	HT	HAT
Liver mass (g)	13.42 ± 2.08	13.03 ± 1.86	11.60 ± 0.95	12.25 ± 1.70	11.26 ± 1.31 ^Ω^
Relative mass of the liver (g/g)	0.031 ± 0.004	0.037 ± 0.004 *	0.031 ± 0.001 ^Ω^	0.034 ± 0.001	0.032 ± 0.003 ^Ω^

Values are presented as mean ± standard deviation based on 8–10 animals per group. C, Control. H, Hypertensive. HA, Hypertensive + Açaí. HT, Hypertensive + Resistance Training. HAT, Hypertensive + Açaí + Resistance Training. *, difference from group C. ^Ω^, difference from group H.

## Data Availability

The data will be shared on reasonable request to the corresponding author.
